# Supplementary material for: Chemical Composition and Antioxidant and Antibacterial Potencies of the Artemisia ordosica Aerial Parts Essential Oil during the Vegetative Period
Source: Molecules. 2022 Dec 14;27(24):8898. doi: 10.3390/molecules27248898 (PMC9781940; doi:10.3390/molecules27248898)
Supplement: Supplementary file 1 [file molecules-27-08898-s001.zip › molecules-2063257-SI.pdf]

Chemical Composition and Antioxidant and Antibacterial Potencies of the *Artemisia ordosica* Aerial Parts Essential Oil during the Vegetative Period

Jize Zhang, Qiang Pan, Xiaoqing Zhang * and Tana

Supplementary Material

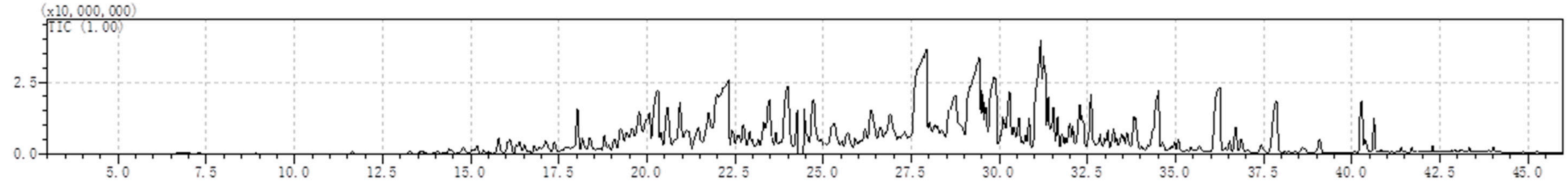

Figure S1. The GC-MS chromatogram of the vegetative-stage essential oil of *A. ordosica*.
